# New models to predict survival of patients with difficult ventilator-weaning diagnosed with community-acquired pneumonia

**DOI:** 10.3389/fmed.2025.1669650

**Published:** 2026-01-12

**Authors:** Shiauyee Chen, Shu-Fen Liao, Wan-Jung Chang, Kin-Fai Ho, Shih-Hsin Hsiao, Shu-Chuan Ho, Jer-Hwa Chang

**Affiliations:** 1 Department of Physical Medicine and Rehabilitation, Wan Fang Hospital, Taipei Medical University, Taipei, Taiwan; 2 School of Respiratory Therapy, College of Medicine, Taipei Medical University, Taipei, Taiwan; 3 Department of Medical Research, Wan Fang Hospital, Taipei Medical University, Taipei, Taiwan; 4 School of Public Health, College of Public Health, Taipei Medical University, Taipei, Taiwan; 5 Division of Pulmonary Medicine, Department of Internal Medicine, Wan Fang Hospital, Taipei Medical University, Taipei, Taiwan; 6 The Jockey Club School of Public Health and Primary Care, The Chinese University of Hong Kong, Sha Tin, Hong Kong, China; 7 Division of Pulmonary Medicine, Department of Internal Medicine, School of Medicine, College of Medicine, Taipei Medical University, Taipei, Taiwan; 8 Division of Pulmonary Medicine, Department of Internal Medicine, Taipei Medical University Hospital, Taipei, Taiwan; 9 Division of Pulmonary Medicine, Department of Internal Medicine, Shuang Ho Hospital, Taipei Medical University, New Taipei City, Taiwan

**Keywords:** community-acquired pneumonia, difficult weaning, respiratory failure, survival, predictive model, respiratory care center, CURB-65 score, pneumonia severity index (PSI)

## Abstract

**Background:**

High mortality is common in mechanically ventilated patients with severe community-acquired pneumonia (CAP). This study sought to create prediction models and determine factors for the pneumonia patients under difficult ventilator weaning in a respiratory care center.

**Methods:**

In total, 353 CAP and hospital-acquired pneumonia (HAP) patients admitted to a respiratory care center (RCC) from January 1, 2015 to December 31, 2017 were included in this retrospective cohort study. Mortality and weaning risks factors were collected and analyzed for validating the prediction models. The study focuses primarily on CAP patients, with HAP data used to external validation.

**Results:**

Among 270 CAP patients in model testing and validation, CURB-65 (Confusion, Uremia, Respiratory rate, Blood pressure, aged ≥65 years) and CUB-65 similarly predicted RCC survival (AUROCs ~65%). Three new RCC survival prediction models incorporated age ≥65, hypotension, BUN >19 mg/dl, ventilator type (replacing respiratory rate), and GCS ≤ 8 (replacing confusion), and either white blood cells (WBCs) or hemoglobin (Hb) were additionally included in model 1 or model 2. In CAP test samples, AUROCs with CAP test sample were 77.51% (model 1), 75.99% (model 2), and 77.97% (model 3). All three new models showed higher AUROCs than CURB-65, with significantly improved predictability in models 1 (*p* = 0.0354) and 3 (*p* = 0.0383). All three models demonstrated satisfactory performance (AUROC ≥ 80%) for predicting RCC survival in the CAP validation sample.

**Conclusions:**

These new models more accurately predicted survival during RCC admission. Furthermore, they offer clinicians a better predictive tool for survival in CAP patients facing difficult ventilator weaning.

## Introduction

Pneumonia is the leading cause of mortality in elderly patients (1, 2) and is classified into community-acquired pneumonia (CAP) and hospital-acquired pneumonia (HAP) (3). CAP is an acute infectious disease accompanied an abnormally increased or decreased white blood cell (WBC) count in a non-hospital setting (3, 4). Severe CAP (SCAP) often results in high mortality (5). About 58%−87% of patients with SCAP were reported to develop respiratory failure and require mechanical ventilation (6).

A 2023 study introduced a mortality risk prediction model for patients with SCAP in intensive care units (ICUs). Lymphocytes, the ratio of partial pressure of oxygen in arterial blood (PaO_2_)/fraction of inspired oxygen (FiO_2_), septic shock, and Acute Physiology and Chronic Health Evaluation (APACHE) II scores were independent risk factors. The model outperformed traditional systems such as APACHE II and Sequential Organ Failure Assessment (SOFA) (7). Another study created a model for elderly SCAP patients, using age, vasopressor use, chronic renal disease, neutrophil and platelet counts, and blood urea nitrogen (BUN) as key risk factors for in-hospital mortality (8).

The 2005 International Consensus Conference defines difficult weaning as when patients have either failed at least three weaning attempts or required more than 7 days of weaning after the first spontaneous breathing trial (SBT). In previous research, risk factors for re-intubation span multiple domains, such as an older age, comorbidities, low hemoglobin (Hb), low albumin, etc (9–12). A retrospective study investigated factors influencing weaning among elderly patients (≥65 years) with CAP, and results showed that patients with emphysematous changes and low albumin concentrations experienced weaning difficulties (11).

Several prediction models help clinicians predict survival for patients with CAP, such as CURB-65 and the Pneumonia Severity Index (PSI) (1). CURB-65 is a simple calculator based on five risk factors including confusion, urea, respiratory rate, systolic blood pressure, and being aged ≥65 years. The PSI contain 20 risk factors that can categorize patients into five groups of mortality risk. These models were validated to predict patient mortality. Factors related to weaning were similar (overlapping) to the factors in these models. CURB-65 was found to be better in predicting admission to an ICU compared to the PSI (13). Since the models have limitations, some studies tried to modify or extend the original models. Liu et al. (14) tried to expand CURB-65 because of low sensitivity for 30-day mortality. The expanded CURB-65 is an objective and accurate scoring system. Sanz et al. (15) added the factor of hypoxemia to CURB-65 for reminding clinicians. The CURB-65 system is easy to apply in clinical practice. However, its application still faces limitations in specific situations, such as in ventilator-using populations. For pneumonia patients under ventilator support, the respiratory rate (RR) is not considered an effective factor for prediction. A review article found that lower Hb levels were significantly associated with increased risks of weaning failure and mortality in critically ill patients (16). An effective model constructed from risk factors in ventilator-using populations could be instrumental in adjusting weaning and treatment plans within the clinical setting.

In 1998 in Taiwan, the National Health Institute established an integrated delivery system for patients who require ventilatory support. Respiratory care centers (RCCs) are specialized units downstream of ICUs that provide effective care for mechanical ventilation (MV)-using patients under difficult weaning (12, 17). Therefore, based on the CURB-65 system, the purposes of this study were to construct new prediction models and understand factors predicting outcomes for pneumonia patients under difficult ventilator weaning in an RCC setting. The study focuses primarily on CAP patients, with HAP data used to external validation.

## Materials and methods

### Study population

This was a retrospective cohort study. Patients (over 20 years of age) admitted to an RCC from January 1, 2015 to December 31, 2017 were recruited. The RCC unit was located in a medical center in northern Taiwan (a single-center study). All patients were transferred from a medical or surgical ICU, a general ward, or the emergency department. The patients with respiratory failure were included if intermittent mandatory ventilation (IMV) or non-invasive ventilator (NIV) lasted longer than 3 weeks and weaning failed. The exclusion criteria included the patients who (1) requested discharge against medical advice after admission to RCC (2) expired within 3 days of admission to the RCC (3) were in the state of shock and used vasopressors within 3 days of admission to RCC. We gained access to the patient list and charts from our secretary department. Patients received a multidisciplinary approach after admission. The study was approved by the Taipei Medical University (TMU)-Joint Institutional Review Board with approval number (N201612048). The study was conducted in accordance with the Declaration of Helsinki.

Of the entire recruited pneumonia population, most patients were identified as having CAP (*N* = 270), and the minority had HAP (*N* = 83). CAP was diagnosed based on a combination of factors. These included clinical symptoms (dyspnea, coughing, fever, fatigue, and changes in mental status) combined with new radiographic infiltrates or abnormal WBC/C-reactive protein levels. Diagnosis required no prior hospitalization in the past 2 weeks (4, 18). HAP was defined as pneumonia occurring 48 h after hospital admission or up to 14 days after discharge (19). In this study, we restricted our cohort to patients with CAP for developing new models to predict their prognosis. The HAP population was further used for external validation to examine the generalizability of the prediction models.

### Definition of covariates and outcomes

CURB-65 was the measure used to predict the survival for pneumonia patients, based on an evaluation of confusion (C), BUN (U), respiratory rate (R), hypotension (B), and being aged ≥65 years for each individual. In our study, we applied the Glascow Coma Scale (GCS) as a substitute for confusion (20). A GCS score of ≤ 10 for patients with the use of invasive MV (IMV) and a GCS score of ≤ 14 for patients with the use of non-invasive ventilation (NIV) were defined as confusion. The criteria of hypotension were systolic blood pressure (SBP) of <90 mmHg or diastolic blood pressure (DBP) of ≤ 60 mmHg. An RR of ≥30/min and a blood BUN level of >19 mg/dl were defined as abnormal. Ventilator use included IMV and NIV. In this study, all enrolled patients were receiving either IMV or NIV. Under these conditions, respiratory rate (RR) does not represent the patient's true respiratory effort because the ventilator influences or determines the observed rate. For this reason, we did not use RR in the modified CURB-65 scoring. Instead, ventilator modality (IMV vs. NIV) was selected as a practical substitute for the RR component. A blood WBC count of <4,000 or >10,000, and Hb of ≤ 10 g/dl were considered an abnormal range. The outcome was survival during RCC admission.

### Statistical analysis

Means and standard deviations (SDs) were used to describe continuous variables, while counts and percentages were employed for categorical variables. To compare distributions of baseline characteristics between RCC survivors and non-survivors, *t*-test and Chi-squared test were applied. A logistic regression was applied for odds ratios (ORs) and the corresponding 95% confidence intervals (CIs) to present associations of demographic and clinical predictors with RCC survival. The CURB-65, CUB-65 (excluding RR from the original CURB-65), and three new prediction models were constructed, and their predictive performances of RCC survival were evaluated using area under receiver operator characteristic curves (AUROCs) (21). DeLong's tests were applied to compare AUROCs between the new prediction models and CURB-65 for assessing their discriminative abilities (22). Besides, decision curve analysis (DCA) (23) and calibration curves were further conducted to comprehensively evaluate model performance (24). To develop RCC prediction models among CAP patients, 4/5 of them were included as the test sample for model training, and 1/5 of them were used for internal validation. The new prediction models developed were presented with nomograms for better visualization. We also evaluated the external validity of the new prediction models based on the predictive performance on 3-month survival or weaning from the ventilator. In addition, the prediction models were validated in HAP patients. We include the HAP patients for external validation because we want to know the possibility of the predictive models applied in this population, since the patients was part of the pneumonia patients in the hospital. The significance level was set to 0.05, and all statistical analyses were performed using SAS software (vers. 9.4; SAS institute, Cary, NC, USA).

## Results

### Study samples

In total, 270 patients were identified as having CAP in this study. To develop a prediction model, 4/5 of the entire study population (*N* = 216) was set as the test sample, and the remaining 1/5 was the validation sample (*N* = 54). Distributions of age, clinical factors (hypotension, BUN, confusion, GCS scores, WBCs, and Hb), and ventilator-related variables (RR and ventilator type) did not significantly differ between the test sample and validation sample, indicating that an unbiased evaluation of the prediction model with the validation sample could be made (Table 1). RCC survival rates of the test sample (87.5%) and validation sample (83.3%) were also similar (*p* = 0.4205; Table 1).

**Table 1:** Demographic characteristics of patients with community-acquired pneumonia (*N* = 270).

**Variables**	**Test sample (*N* = 216)**	**Validation sample (*N* = 54)**	***p*-value**
**Age** ≥**65 years**			
No	18 (8.33)	8 (14.81)	0.1487
Yes	198 (91.67)	46 (85.19)	
**Hypotension** ^a^			
No	89 (41.20)	25 (46.30)	0.4980
Yes	127 (58.80)	29 (53.70)	
**RR** ≥**30/min**			
No	183 (84.72)	50 (92.59)	0.1325
Yes	33 (15.28)	4 (7.41)	
**BUN** >**19 mg/dl**			
No	49 (22.69)	15 (27.78)	0.4312
Yes	167 (77.31)	39 (72.22)	
**Confusion** ^b^			
No	53 (24.54)	12 (22.22)	0.7219
Yes	163 (75.46)	42 (77.78)	
**GCS** ≤ **8**			
No	175 (81.02)	41 (75.93)	0.4027
Yes	41 (18.98)	13 (24.07)	
**Ventilator type**			
IMV	96 (44.44)	22 (40.74)	0.6236
NIV	120 (55.56)	32 (59.26)	
**WBCs**<**4,000 or WBCs** >**10,000**			
No	112 (51.85)	24 (44.44)	0.3302
Yes	1014 (48.15)	30 (55.56)	
**Hb** ≤ **10 g/dl**			
No	96 (44.44)	17 (31.48)	0.0841
Yes	120 (55.56)	37 (68.52)	
**RCC survival**			
No	27 (12.50)	9 (16.67)	0.4205
Yes	189 (87.50)	45 (83.33)	

RR, respiratory rate; BUN, blood urea nitrogen; WBCs, white blood cells; Hb, hemoglobin; RCC, respiratory care center.

^a^The definition of hypotension was systolic blood pressure of <90 mmHg or diastolic blood pressure of ≤ 60 mmHg.

^b^The definition of confusion was a Glascow Coma Scale (GCS) score of ≤ 10 when invasive mechanical ventilation (IMV) was used and a GCS score of ≤ 14 when IMV and when non-invasive ventilation (NIV) was used.

### Ability of CURB-65 to predict RCC survival

In Supplementary Table 1, we compared the ability of CURB-65 and CUB-65 (with RR excluded from CURB-65) to predict RCC survival. AUROCs for CURB-65 and CUB-65 among CAP patients were 65.0% (95% CI = 56.7%, 73.3%) and 64.8% (95% CI = 57.0%, 72.5%), respectively. No significant difference was detected when applying CURB-65 and CUB-65 to predict RCC survival (*p* = 0.8425), suggesting that RR was not a necessary factor for the prediction model among the CAP population. We also assessed whether the new prediction models obtained better performances for predicting RCC survival compared to CURB-65 or CUB-65.

### Associated factors for the new RCC survival prediction models

In the test sample, associations between clinical characteristics and RCC survival were examined (Table 2). Patients older than 65 years had a lower RCC survival rate (86.36%) compared to younger patients (100.0%). Patients with hypotension, and abnormal BUN, WBCs, and Hb values exhibited lower survived during RCC hospitalization compared to healthier patients. The type of ventilator used was significantly associated with RCC survival, at 80.8% for NIV and 95.8% for IMV. Patients with a confused status or a GCS score of ≤ 8 were seen to have lower survival rates than their comparators, and the difference in survival rates was statistically significant for GCS scores of ≤ 8.

**Table 2:** Associations between clinical characteristics and RCC survival in the test sample (*N* = 216).

**Variables**	**RCC survival (*N* = 189)**	**RCC non-survival (*N* = 27)**	***p*-value**
**Age** ≥**65 years**			
No	18 (100.00)	0 (0.00)	0.1375
Yes	171 (86.36)	27 (13.64)	
**Hypotension** ^a^			
No	81 (91.01)	8 (8.99)	0.1915
Yes	108 (85.04)	19 (14.96)	
**BUN** >**19 mg/dl**			
No	46 (93.88)	3 (6.12)	0.1247
Yes	143 (85.63)	24 (14.37)	
**Confusion** ^b^			
No	47 (88.68)	6 (11.32)	0.7651
Yes	142 (87.12)	21 (12.88)	
**GCS** ≤ **8**			
No	157 (89.71)	18 (10.29)	**0.0421**
Yes	32 (78.05)	9 (21.95)	
**Ventilator type**			
IMV	92 (95.83)	4 (4.17)	**0.0009**
NIV	97 (80.83)	23 (19.17)	
**WBCs**<**4,000 or WBCs** >**10,000**			
No	102 (91.07)	10 (8.93)	0.0996
Yes	87 (83.65)	17 (16.35)	
**Hb** ≤ **10 g/dl**			
No	85 (88.54)	11 (11.46)	0.6788
Yes	104 (86.67)	16 (13.33)	

^a^The definition of hypotension was systolic blood pressure of <90 mm-Hg or diastolic blood pressure of ≤ 60 mm-Hg.

^b^The definition of confusion was a Glascow Coma Scale (GCS) score of ≤ 10 when invasive mechanical ventilation (IMV) was used and a GCS score of ≤ 14 when IMV and when non-invasive ventilation (NIV) was used.

Footnote to indicate the meaning of the bold values: BUN, blood urea nitrogen; GCS, Glascow Coma Scale; IMV, invasive mechanical ventilation; NIV, non-invasive ventilation; WBCs, white blood cells; Hb, hemoglobin.

### Constructing new RCC survival prediction models

**Figure 1:**
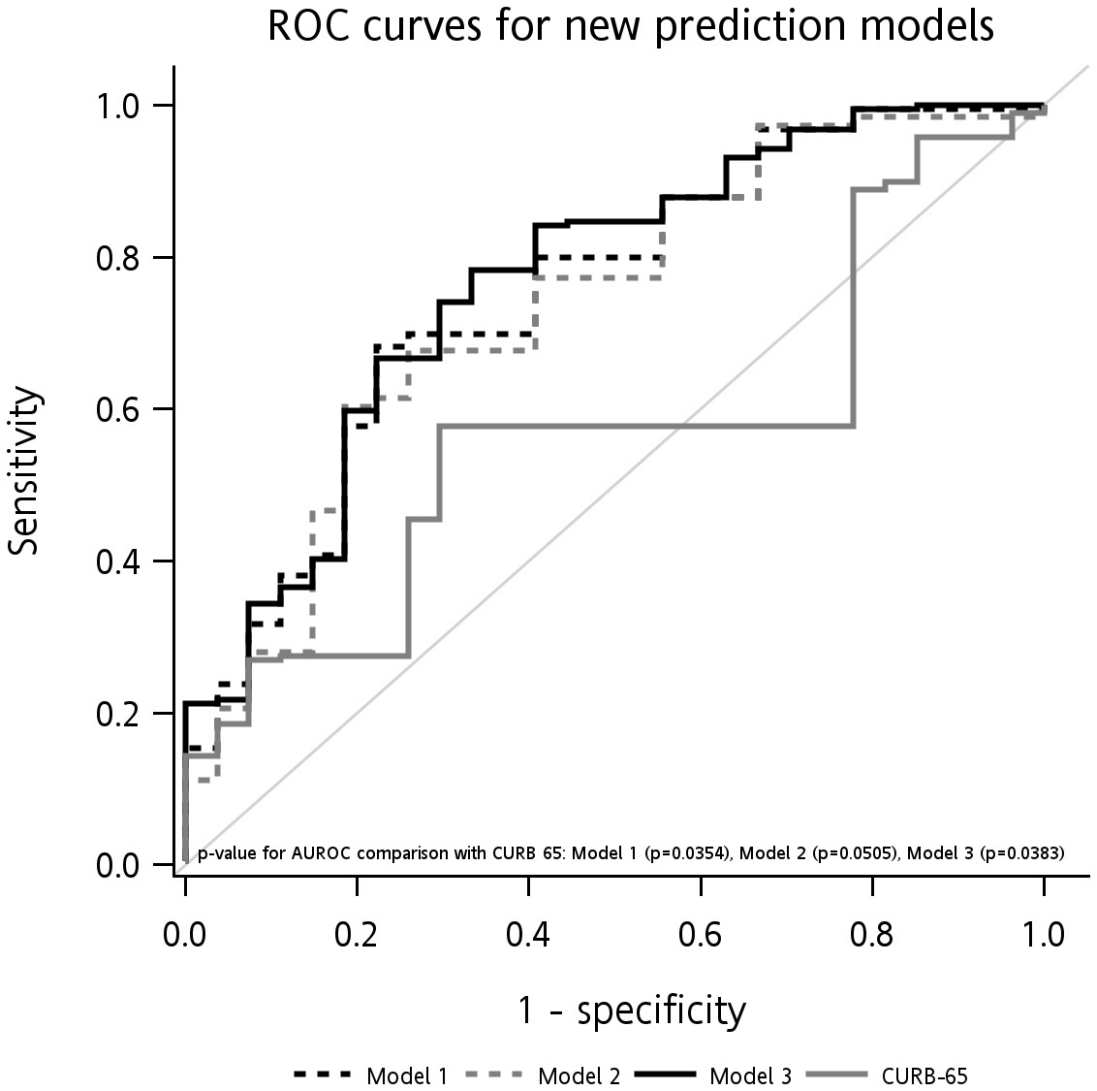
ROC curves for three new prediction models in test sample and their comparisons in RCC survival predictability with CURB-65. Model 1 included elementary variables (aged ≥65 years, hypotension, BUN >19 mg/dl, ventilator type, GCS score of ≤ 8), and abnormal WBCs (<4,000 or >10,000). Model 2 included elementary variables, and abnormal Hb ( ≤ 10 g/dl). Model 3 included elementary variables, and both of abnormal WBCs and Hb.

Table 3 and Figure 1 presents data of three new prediction models for RCC survival among patients with CAP. Nomograms for three new prediction models were also presented in Supplementary Figure 1. All of the models included the elements of the CURB-65 of being aged ≥65 years, hypotension, BUN >19 mg/dl, ventilator type (replacing RR), and a GCS score of ≤ 8 (replacing confusion). Either WBCs or Hb were additionally included in model 1 or model 2. And model 3 was an integrated model including all of the aforementioned variables. AUROCs for the test sample were 77.51% (95% CI = 67.66%, 87.36%) for model 1, 75.99% (95% CI = 65.69%, 86.30%) for model 2, and 77.97% (95% CI = 68.10%, 87.85%) for model 3. Compared to CURB-65 for predictions, all three models had higher AUROCs, and the increases in predictability were significant in models 1 (*p* = 0.0354) and 3 (*p* = 0.0383). All the comparisons were consistently presented when DCA and calibration analyses were performed, showing better performance with three new prediction models than CURB-65 (Supplementary Figure 2). All three models maintained satisfactory performance (at least 80% for AUROCs) to predict RCC survival in the validation sample.

**Table 3:** New prediction model for RCC survival among patients with community-acquired pneumonia.

**Variables**	**Model 1**		**Model 2**		**Model 3**	
	**OR (95% CI)**	***p*** **-value**	**OR (95% CI)**	***p*** **-value**	**OR (95% CI)**	***p*** **-value**
**Age** ≥**65 years**						
No	–	0.9747	–	0.9750	–	0.9748
Yes	Ref.		Ref.		Ref.	
**Hypotension** ^a^						
No	1.59 (0.62, 4.07)	0.3305	1.60 (0.63, 4.07)	0.3237	1.64 (0.64, 4.22)	0.3050
Yes	Ref.		Ref.		Ref.	
**BUN** >**19 mg/dl**						
No	2.20 (0.59, 8.17)	0.2394	2.13 (0.58, 7.86)	0.2574	2.14 (0.57, 8.00)	0.2572
Yes	Ref.		Ref.		Ref.	
GCS ≤ 8
No	2.49 (0.94, 6.63)	0.0671	2.38 (0.91, 6.24)	0.0787	2.40 (0.90, 6.45)	0.0814
Yes	Ref.		Ref.		Ref.	
**Ventilator type**						
IMV	Ref.		Ref.		Ref.	
NIV	0.17 (0.06, 0.52)	0.0020	0.17 (0.05, 0.51)	0.0017	0.16 (0.05, 0.50)	0.0017
**WBCs**<**4,000 or WBCs** >**10,000**						
No	2.15 (0.89, 5.19)	0.0890			2.25 (0.92, 5.48)	0.0754
Yes	Ref.				Ref.	
**Hb** ≤ **10 g/dl**						
No			1.30 (0.54, 3.13)	0.5553	1.44 (0.59, 3.52)	0.4264
Yes			Ref.		Ref.	
AUROC (%; test sample)	77.51 (67.66, 87.36)		75.99 (65.69, 86.30)		77.97 (68.10, 87.85)	
*p*-Value^b^	0.0354		0.0505		0.0383	
AUROC (%; validation sample)	86.54 (75.82, 97.27)		81.36 (68.69, 94.02)		86.05 (75.15, 96.94)	

OR, odds ratio; CI, confidence interval; BUN, blood urea nitrogen; GCS, Glascow Coma Scale; IMV, invasive mechanical ventilation; NIV, non-invasive ventilation; WBCs, white blood cells; Hb, hemoglobin.

^a^The definition of hypotension was systolic blood pressure of <90 mm-Hg or diastolic blood pressure of ≤ 60 mm-Hg.

^b^*p*-value for comparing with CURB-65 in the test sample. Area under the receiver operating characteristic curve (AUROC; %) for CURB-65 in the test sample: 66.09 (55.98, 76.20).

### External validation for 3-month survival as prediction models

Table 4 presents results of the RCC survival prediction models applied to extension analyses. External validation targeted the predictability of 3-month survival and weaning among patients with CAP. We also performed validation in HAP patients. For 3-month survival, the new prediction models did not work much better than CURB-65 among all patients with CAP, with an AUROC of about 70%. The new prediction models and CURB-65 presented worse abilities to predict weaning, with as low an AUROC of about 60%. Compared to the predictability of new models on RCC survival, those for 3-month survival and for weaning were both reduced. When the three prediction models were validated with the HAP sample, AUROCs increased to as high as 80%, but the performances were not significantly better than that of CURB-65.

**Table 4:** External validation of the prediction model.

**Models**	**AUROC (%; total sample)**	***p*-value^a^**
**3-month survival among patients with community-acquired**		
**pneumonia (*****N*** = **219)**		
CURB-65	71.27 (64.55, 77.99)	–
CUB-65	70.98 (64.31, 77.65)	0.5850
Model 1	69.54 (62.60, 76.48)	0.4908
Model 2	71.67 (64.84, 78.50)	0.8693
Model 3	71.89 (65.09, 78.68)	0.8176
**Weaning**^b^ **among patients with community-acquired**		
**pneumonia (*****N*** = **270)**		
CURB-65	62.83 (56.19, 69.47)	–
CUB-65	62.27 (55.58, 68.96)	0.7505
Model 1	65.61 (58.75, 72.48)	0.4573
Model 2	64.29 (57.54, 71.03)	0.6712
Model 3	65.77 (58.85, 72.70)	0.4326
**RCC survival among patients with hospital-acquired**		
**pneumonia (*****N*** = **83)**		
CURB-65	80.26 (68.34, 92.19)	–
CUB-65	77.82 (64.37, 91.27)	0.6491
Model 1	87.31 (76.33, 98.30)	0.4466
Model 2	84.96 (74.38, 95.55)	0.5631
Model 3	87.78 (77.79, 97.77)	0.4047

AUROC, area under the receiver operating characteristic curve; CURB-65, confusion, uremia, ventilator type, blood pressure, aged ≥65 years; CUB-65, confusion, uremia, blood pressure, aged ≥ 65 years; RCC, respiratory care center.

^a^*p*-value for comparing with CURB-65 in the total sample.

^b^Criteria for weaning: success in weaning was defined as 5 days of complete liberation from the ventilator.

## Discussion

The models we built are more appropriate for predicting short-term survival in RCCs (for an average of 18.39 days). We could not predict the long-term survival of these patients, such as 1-year mortality. Hung conducted a series of studies focused on RCC settings in Taiwan. His study, specifically, investigated factors affecting the survival of patients with prolonged mechanical ventilation (MV) in these RCCs. The 1- and 5-year survival rates reported were 24.3 and 14.6%, respectively. Patients who successfully weaned and received a tracheostomy showed better survival outcomes. Factors related to patient outcome (ward mortality vs. discharged home) included end-stage renal disease (ESRD), receipt of a tracheostomy, and the presence of four or more morbidities (25, 26).

For our population, the new prediction models and CURB-65 presented poor abilities to predict weaning in CAP patients, with AUROCs of only about 60%. In a 2021 study, patients who failed extubation presented with higher mean CURB-65 scores (4.36) compared to their counterparts (3.88). Patients who scored 5 on the CURB-65 were significantly more likely to have failed the extubation process compared to those who scored 3 or 4 (27). In that study, there were different populations with high Burns Wean Assessment Program (BWAP) scores of ≥50. Neither CURB-65 nor our new models appear to be broadly predictive indicators for successful weaning in certain CAP patients.

In a study, some risk assessment scores including CURB-65 were used to predict all-cause 30-day mortality rate of 223 HAP patients. The PSI, CURB-65, SOFA, APACHE II, and qSOFA scores were significantly higher in non-survivors than in survivors. The CURB-65 score presented an AUROC of 0.744. This finding was consistent with our study. Notably, our CURB-65 scores showed better discriminative ability for survival among HAP patients, with AUROCs of up to 80%. The AUROCs of our new models performed even better than CURB-65, indicating the successful inclusion of effective predictive factors in our new models. However, the small sample size of HAP patients in our study resulted in a lack of power to detect differences in discrimination between the new models and CURB-65 (28).

For our population, fewer patients with GCS scores of ≤ 8, compared to the confusion status, survived, and a significant difference in the survival rate was detected. This was consistent with a previous study in that patients with GCS scores of 9–12 had a 99.3% faster recovery rate relative to patients with GCS scores of ≤ 8 [adjusted hazard ratio (AHR): 1.993; 95% confidence interval (CI): (1.358–2.924)]; and critically ill patients with GCS scores of 13–15 had two-fold longer recovery times than patients with GCS scores of ≤ 8 [AHR: 2.451; 95% CI: (1.483–4.051)] (2, 20, 29). So It was reasonable to use GCS scale 8 as a simplified cutoff point of GCS data grouping for outcome analysis. This indicates that the GCS could serve as a substitute for the confusion status. A GCS score of ≤ 8 could also be an easier cut-point for a new scoring system in ventilator-using populations.

RR is difficult to interpret once mechanical ventilation begins. In assisted or controlled IMV, the displayed rate often reflects the ventilator's cycling rather than the patient's intrinsic drive, as described by Yang and Tobin (30). Classic mechanical ventilation principles further emphasize that RR becomes machine-dependent after ventilatory assistance begins (31). For NIV, pressure support can change the patient's breathing pattern and make RR unstable, which Brochard et al. (32) have shown. Because of these limitations, ventilator modality gives a clearer indication of respiratory failure severity than RR in this population.

Lower Hb levels are associated with poorer weaning outcomes in MV patients. Low Hb levels impair oxygen delivery and respiratory muscle function. Consequently, this leads to an overall poor physical condition, which hinders the patient's ability to breathe independently after extubation. Lower Hb levels were found to be a significant predictor of higher mortality rates. Our previous studies found that lower Hb was associated with worse outcomes such as extubation failure and prolonged ventilation (33, 34).

A systematic review and meta-analysis found no significant differences in mortality prediction between the CURB-65, CRB-65, and PSI scores for CAP (35). Conversely, complete blood counts and differential leukocyte counts (CBCs/DCs) are commonly used for unidentified infections. The Blood Culture Prediction Index (BCPI), which combines CBC and DC, outperformed CURB-65 in predicting 30-day and in-hospital mortality. After integrating the CURB-65 and BCPI models, study results found a higher AUC than that of CURB-65 alone (36). Despite similar overall performances, each score has unique characteristics for different clinical settings.

The current limitations are as follows:(1) This was a retrospective, single-center study, which limits the external validity and may introduce bias. (2) The small sample size of HAP patients (*n* = 83) in the external validation cohort may affect the reliability of the results for this group. (3) The lack of external validation in a broader, multicenter CAP population is crucial to assess the generalizability of the models in different clinical settings. (4) Although we reviewed pre-existing pulmonary comorbidities from medical histories, we lacked the data of lung function tests. These prior pulmonary pathologies may significantly influence the clinical course and prognosis of patients managed with mechanical ventilation.

Future studies should apply the three predictive models in a prospective, multicenter study design to CAP or HAP populations. This would be helpful in establishing the generalizability of the models across different regions. If our new models are widely validated in further studies, we might design an outcome scoring system for patients under ventilator use in RCC. It wound possibly serve for a personalized weaning protocol and management, and translocation strategy. And this might further provide a specific intervention or decision planning in a RCC setting.

## Conclusions

Our new models demonstrated a higher predictive accuracy for survival during RCC admission, compared to existing scores, giving clinicians a potentially more-effective tool for CAP patients undergoing difficult ventilator weaning. This improvement suggests that new models might be better suited to accurately predict outcomes in specific populations.

## Data Availability

The original contributions presented in the study are included in the article/Supplementary material, further inquiries can be directed to the corresponding authors.
