# Supplementary material for: New models to predict survival of patients with difficult ventilator-weaning diagnosed with community-acquired pneumonia
Source: Front Med (Lausanne). 2026 Jan 12;12:1669650. doi: 10.3389/fmed.2025.1669650 (PMC12832357; doi:10.3389/fmed.2025.1669650)

**Supplementary table 1. AUROC of CURB-65 for predicting RCC survival**

|  | CURB-65 | CUB-65 |
| --- | --- | --- |
| Age ≥ 65 years | V | V |
| Hypotension ^a^ | V | V |
| RR ≥ 30/min | V |  |
| BUN > 19 mg/dL | V | V |
| Confusion ^b^ | V | V |
| **AUROC (%) (among all CAP patients)** | 65.00 (56.74, 73.27) | 64.77 (57.01, 72.53) |
| **p-value ^c^** | 0.8425 | |

^a^ The definition of hypotension was systolic blood pressure of < 90 mm-Hg or diastolic blood pressure of ≤ 60 mm-Hg.

^b^ The definition of confusion was a Glascow Coma Scale (GCS) score of ≤ 10 when invasive mechanical ventilation (IMV) was used and a GCS score of ≤14 when IMV and when non-invasive ventilation (NIV) was used..

^c^ p-value for comparing CURB-65 and CUB-65

**Supplementary figure 1. Nomograms for new RCC survival prediction models**

1. Model 1 (B) Model 2


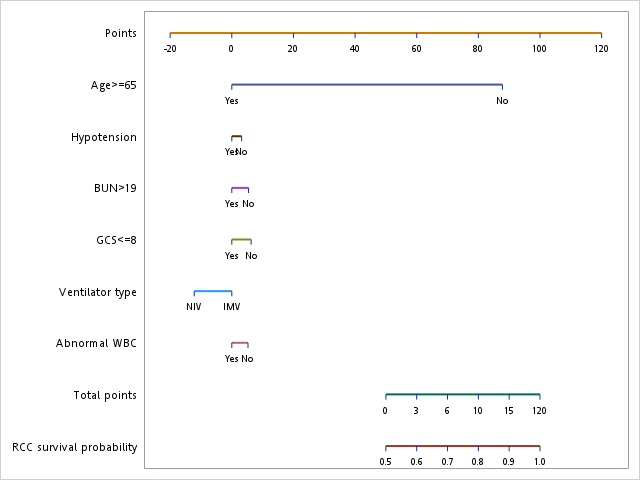

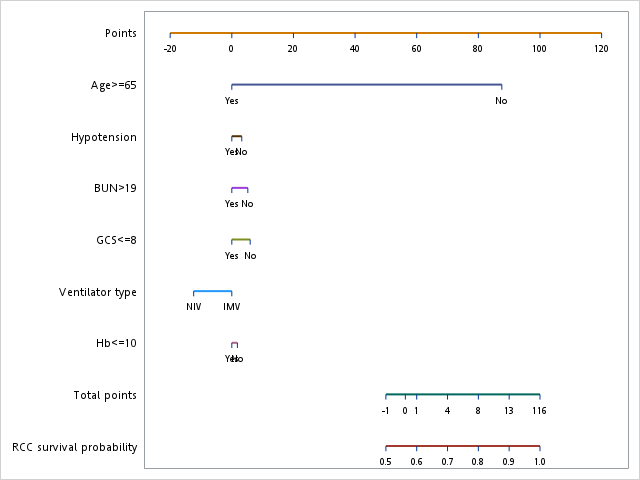


Abnormal WBC: WBCs < 4000 or WBCs > 10,000

1. Model 3


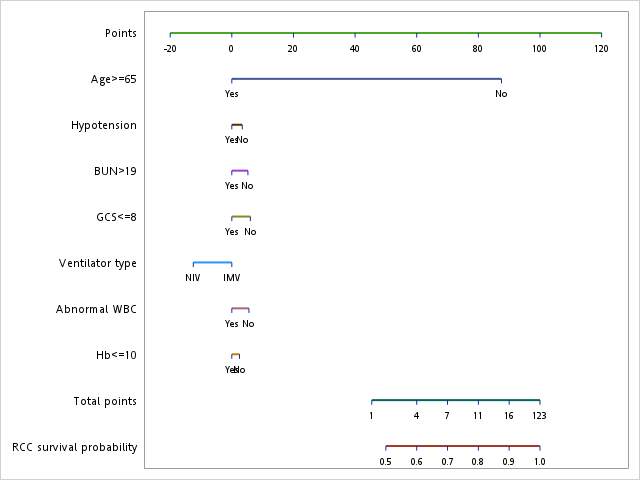


Abnormal WBC: WBCs < 4000 or WBCs > 10,000

**Supplementary figure 2. Decision curve analysis (A) and calibration curves (B) for new RCC survival prediction models**

1. Decision curve analysis (B) Calibration curves


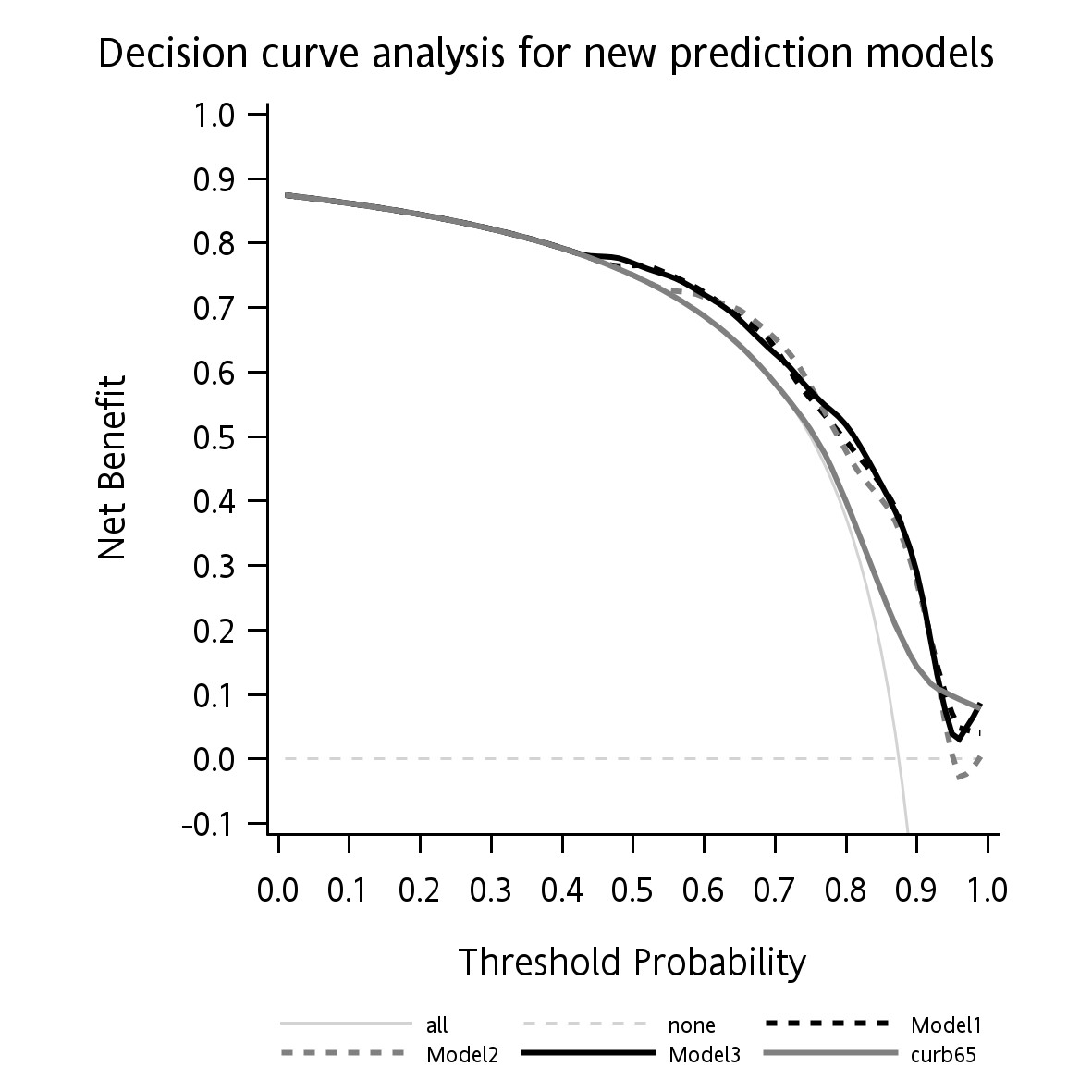

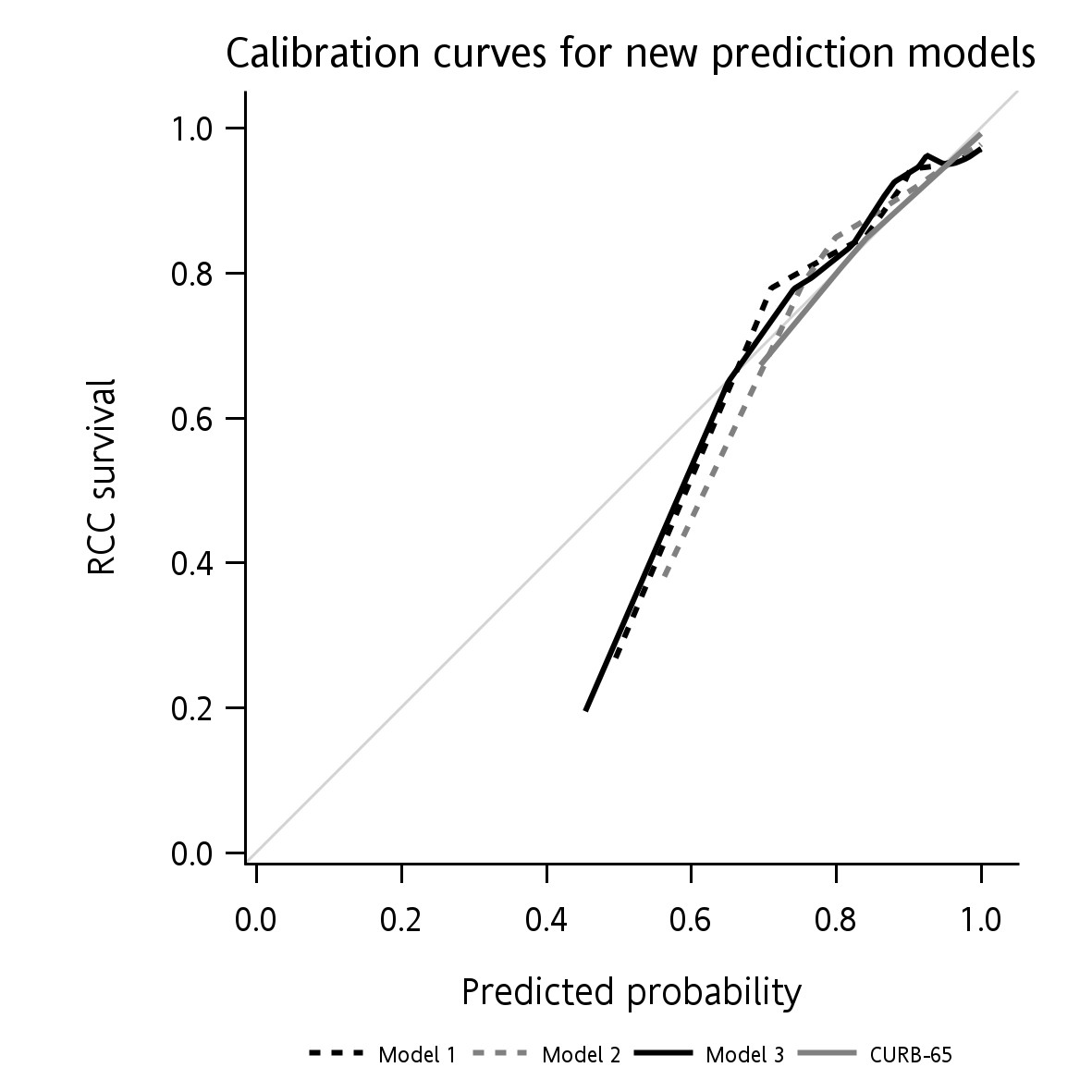

Supplement: Supplementary file 1 [file Supplementary_file_1.docx]
